# Poly(3-hydroxybutyrate) production from ricotta whey by *Cupriavidus necator*: effects of coffee oil and oxidative stress

**DOI:** 10.1007/s00253-026-13933-8

**Published:** 2026-06-26

**Authors:** Angela Longo, Andrea Torreggiani, Luca Sconosciuto, Michela Verni, Vito Emanuele Carofiglio, Domenico Centrone, Marianna Villano, Gaia Salvatori, Erica Pontonio, Marco Montemurro, Carlo G. Rizzello

**Affiliations:** 1 Department of Environmental Biology, Sapienza University of Rome, 00185 Rome, Italy; 2 Department of Soil, Plant and Food Science, University of Bari, 70126 Bari, Italy; 3 EggPlant Srl, 00198 Rome, Italy; 4 Department of Human Science and Quality of Life Promotion, San Raffaele University, 00166 Rome, Italy; 5 Department of Chemistry, Sapienza University of Rome, 00185 Rome, Italy; 6 Research Center for Applied Sciences to the Safeguard of Environment and Cultural Heritage (CIABC), Sapienza University of Rome, 00185 Rome, Italy; 7 Institute of Sciences of Food Production, (CNR-ISPA), National Research Council of Italy, 70126 Bari, Italy

**Keywords:** PHB, *Cupriavidus necator*, Whey, Oxidative stress, Fed-batch fermentation

## Abstract

**Abstract:**

In this study, ricotta cheese exhausted whey (RCEW), an abundant dairy by-product, was evaluated as a low-cost substrate for poly(3-hydroxybutyrate) (PHB) production by *Cupriavidus necator* DSM 428 through an integrated process strategy combining substrate conditioning, enzymatic lactose hydrolysis, oxidative stress modulation, and cultivation under controlled bioreactor conditions. Shake-flask experiments demonstrated that RCEW supported efficient biomass formation (1.60 g L⁻^1^) and high intracellular PHB accumulation (71.4% of dry cell weight, DCW), while supplementation with coffee oil extract increased biomass up to 4.41 g L⁻^1^ and PHB titer to 2.87 g L⁻^1^. Controlled oxidative stress further enhanced polymer accumulation, with hydrogen peroxide addition (5.0 mmol L⁻^1^ at 24 h) promoting intracellular PHB contents up to 80.8% DCW. Cultivation in a stirred-tank bioreactor confirmed process robustness, and fed-batch cultivation resulted in PHB concentrations up to 3.85 g L⁻^1^ (77.9% DCW) using a chlorine-free downstream recovery protocol. The recovered polymer consisted exclusively of 3-hydroxybutyrate units and exhibited a high–molecular weight (876 kDa). Overall, these results demonstrate the feasibility of producing high-quality PHB from RCEW using a non-recombinant *C. necator* strain, supporting the development of sustainable bioprocesses for dairy by-product valorization.

**Key points:**

- *Thermally conditioned ricotta cheese exhausted whey supports efficient PHB production by C. necator*
- *Coffee oil supplementation enhances biomass formation whereas oxidative stress promotes PHB accumulation*
- *Fed-batch cultivation and chlorine-free recovery support process efficiency and sustainability*

**Supplementary Information:**

The online version contains supplementary material available at https://doi.org/10.1007/s00253-026-13933-8.

## Introduction

The widespread production and use of petroleum-based plastics represent a major global environmental concern. Conventional plastics are derived from non-renewable fossil resources and are characterized by extremely slow degradation rates, leading to their accumulation in terrestrial and aquatic ecosystems for decades or even centuries (Zhang et al. 2022; Wei and Zimmermann 2017; Longo et al. 2024). In addition, fossil-based plastic production is associated with significant greenhouse gas emissions, contributing substantially to the overall carbon footprint of the chemical industry (Hectors et al. 2025; Weldon and Euler 2025; Zhu et al. 2025). These issues have driven increasing interest in biodegradable and bio-based alternatives capable of reducing both fossil resource dependence and plastic pollution (Zhang et al. 2022; Rosenboom et al. 2022).

Among bio-based alternatives, microbial bioplastics such as polyhydroxyalkanoates (PHAs) are regarded as particularly promising. Several classes of bio-based plastics have been proposed as alternatives to conventional petroleum-derived polymers, including starch-based plastics, cellulose-derived materials, polylactic acid (PLA), polybutylene succinate (PBS), and polyhydroxyalkanoates (PHAs). Although many of these materials are produced from renewable resources, they often exhibit limitations related to end-of-life management, industrial compostability requirements, limited biodegradation under natural environmental conditions, or dependence on dedicated agricultural feedstocks. In contrast, PHAs are uniquely synthesized by microorganisms from renewable carbon sources and are fully biodegradable in a wide range of terrestrial and aquatic environments. Their combination of biodegradability, biocompatibility, and production from industrial by-products and waste streams makes them particularly attractive candidates for the development of circular and sustainable plastic value chains (Rosenboom et al. 2022). PHAs can be accumulated by numerous bacterial species under conditions of carbon excess and nutrient limitation. Well-known PHA-producing microorganisms include *Cupriavidus necator*, *Azohydromonas lata*, *Pseudomonas putida*, *Bacillus megaterium*, and several halophilic archaea such as *Haloferax mediterranei*, which have been extensively investigated for both fundamental studies and industrial-scale biopolymer production (Morlino et al. 2023; Zhang et al. 2022).

In particular, poly(3-hydroxybutyrate) (PHB) has been extensively investigated as a substitute for conventional thermoplastics due to its mechanical and thermal properties comparable to polypropylene combined with full biodegradability and biocompatibility (Hectors et al. 2025; Zhang et al. 2022).

Despite these advantages, the industrial implementation of PHAs remains limited by high production costs. Microbial fermentation processes for PHB are still significantly more expensive than petrochemical plastic production, with carbon substrates accounting for approximately 50–60% of total production costs (Zhang et al. 2022). Therefore, improving economic feasibility requires both optimization of microbial productivity and the use of low-cost, renewable carbon sources, preferably derived from industrial by-products or waste streams (Morlino et al. 2023; Weldon and Euler 2025).

In this context, *Cupriavidus necator* has emerged as one of the most promising microbial platforms for PHA production. This Gram-negative bacterium is characterized by remarkable metabolic versatility and has been extensively studied at physiological and metabolic levels (Morlino et al. 2023; Weldon and Euler 2025). The availability of advanced metabolic engineering tools and well-established cultivation strategies further consolidates *C. necator* as a robust chassis for converting low-value carbon sources into high-value biodegradable polymers (Hectors et al. 2025; Zhang et al. 2022).

Various low-cost feedstocks have been investigated for PHA production, including lignocellulosic hydrolysates, crude glycerol from biodiesel manufacture, molasses, waste vegetable oils, and mixed food-processing residues. Although these substrates can support microbial growth and polymer accumulation, their industrial exploitation is often challenged by compositional variability, the presence of inhibitory compounds, or the need for extensive pretreatment and downstream conditioning. In contrast, dairy by-products such as whey and ricotta cheese exhausted whey are characterized by high carbohydrate content, widespread availability, and liquid-state processability, making them particularly attractive substrates for microbial fermentations. Moreover, their high organic load represents a significant environmental burden if discharged untreated, further supporting their valorization within circular bioeconomy strategies (Zhang et al. 2022; Pires et al. 2021). Cheese whey is generated in amounts of approximately 9–10 L per kilogram of cheese produced and accounts for 85–95% of the original milk volume, retaining around 55% of milk nutrients, mainly as lactose (Pires et al. 2021). At the global scale, annual whey and whey permeate production has been estimated at approximately 200 × 10⁶ tons, highlighting the enormous availability of this agro-industrial by-product (El-Aidie and Khalifa 2024). Improper disposal of whey represents a significant environmental burden due to its high biological and chemical oxygen demand (Pires et al. 2021).

An even less valorized by-product is ricotta cheese exhausted whey (RCEW), generated during the production of whey cheeses such as ricotta. RCEW represents more than 90% of the original whey volume after protein recovery and is characterized by high residual lactose content combined with low protein and fat concentrations, resulting in very high carbon content in terms of BOD (Biochemical Oxygen Demand) and COD (Chemical Oxygen Demand) values if discharged untreated (Pires et al. 2021). Recent studies on alternative PHA-producing microorganisms have confirmed the consistent lactose content of RCEW and highlighted key process-related aspects such as compositional variability and the importance of limiting pretreatment severity to maintain economic feasibility (Raho et al. 2020; Longo et al. 2025). Although these studies were not conducted on *C. necator*, they support the suitability of RCEW as an abundant and inexpensive substrate for microbial biopolymer production.

Since the protein content of whey-derived streams can strongly influence the carbon-to-nitrogen (C/N) ratio and consequently PHA accumulation, different thermal treatments and substrate formulations were evaluated. In particular, pasteurization and sterilization were used not only to reduce microbial contamination but also to modulate protein availability through heat-induced protein precipitation, allowing the preparation of substrates with different nutritional profiles and C/N ratios (Longo et al. 2025).

In addition to dairy-derived carbon sources, waste lipids have attracted considerable attention as co-substrates for PHA production because their high carbon and energy content can support both biomass formation and polymer biosynthesis. Among them, coffee-processing residues represent an abundant and largely underutilized waste stream. Coffee oil extracted from spent coffee materials contains significant amounts of fatty acids that can be converted into acetyl-CoA and reducing equivalents, key precursors for PHA synthesis. Previous studies (Bhatia et al. 2018) have demonstrated the feasibility of producing PHA from coffee-derived oils using *Cupriavidus necator*-related strains, highlighting their potential as low-cost substrates within circular bioeconomy frameworks. Therefore, in the present study, coffee oil extract was evaluated as a complementary carbon source to investigate whether its addition could enhance biomass formation and PHB production during cultivation on ricotta cheese exhausted whey.

In addition to substrate optimization, controlled oxidative stress has recently emerged as a promising strategy to enhance PHA accumulation in *Cupriavidus necator* by modulating intracellular redox balance and precursor availability, suggesting its potential as an additional process-level lever for improving PHB productivity (Obruca et al. 2021).

**Fig. 1:**
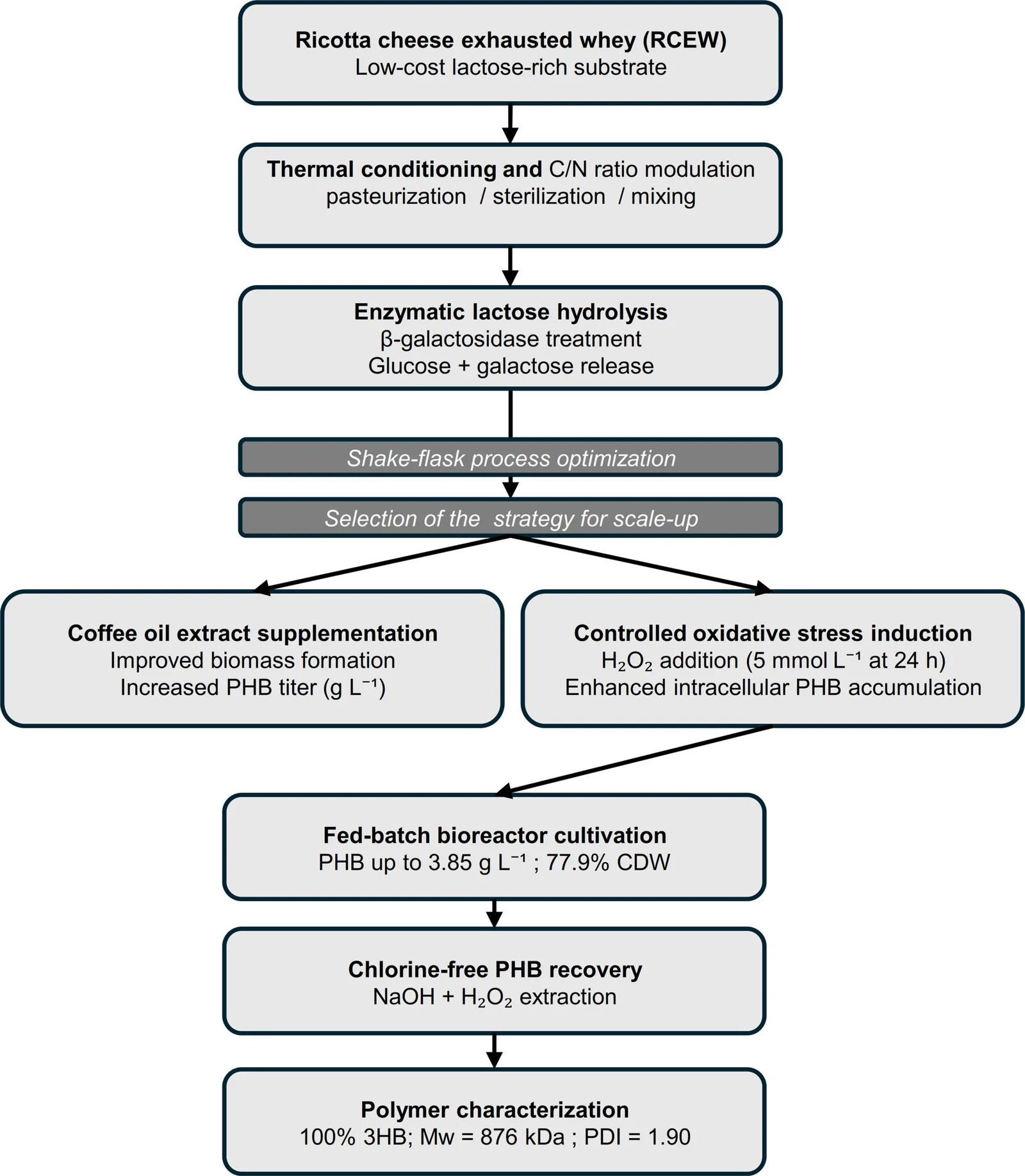
Schematic overview of the integrated upstream and downstream process optimization strategy for PHB production from ricotta cheese exhausted whey (RCEW) using *Cupriavidus necator* DSM 428. Following substrate conditioning and enzymatic lactose hydrolysis, two alternative approaches were evaluated at shake-flask scale, namely supplementation with coffee oil extract (EOC) and controlled oxidative stress induction. Oxidative stress applied at 24 h was selected for subsequent fed-batch bioreactor cultivation, combined with chlorine-free downstream recovery and physicochemical characterization of the resulting polymer

Based on these premises, the present work aims to optimize a biotechnological process for poly(3-hydroxybutyrate) (PHB) production from ricotta cheese exhausted whey (RCEW) using *Cupriavidus necator*, with the dual objective of maximizing microbial productivity while preserving process simplicity and industrial efficiency through minimal substrate pretreatment, limited nutrient supplementation, and streamlined downstream processing. The overall experimental strategy adopted in this study is schematically summarized in Fig. 1.

## Materials and methods

### Microorganism and culture conditions

The bacterial strain *Cupriavidus necator* DSM 428 was purchased from DSMZ collection (Braunschweig, Germany) and cultivated in nutrient broth DSMZ Medium 1, containing peptone, 5.0 g L^−1^; meat extract 3.0 g L^−1^(final pH 7.0). *C. necator* DSM 428 was propagated daily using a 10% (v/v) inoculum coming from a 24 h-refreshed culture and incubated in a 300 ml Erlenmeyer flask at 30 °C for 24 h under constant shaking at 180 rpm. Cell growth in the culture Medium 1 was monitored spectrophotometrically (PerkinElmer mod. Lambda 20) by measuring cell density at 620 nm and by microscopic count obtained using the Thoma cell counting chamber.

### Ricotta cheese exhausted whey characterization

RCEW was provided by EggPlant S.r.l. (Rome, Italy) and originated from dairy processing facilities located in the Apulia region (Italy). The substrate was transported under refrigerated conditions (4 ± 2 °C) and delivered within 24 h of production. Upon arrival, RCEW was characterized and subsequently stored at − 20 °C until use as a cultivation substrate for *C. necator* DSM 428. The pH of RCEW was measured using a FiveEasy Plus pH meter (Mettler-Toledo, Columbus, OH, USA).

Total nitrogen (TN) content was determined according to the ISO 8968–1 Kjeldahl method (ISO/FIL 2001), applying a nitrogen-to-protein conversion factor of 6.38 to estimate total protein concentration. The concentrations of individual sugars (glucose, galactose, and lactose) were quantified by HPLC analysis using an ÄKTA purifier system (GE Healthcare) equipped with a Spherisorb-5-NH₂ column (4.6 × 250 mm; Waters, Sesto San Giovanni, Italy) and a refractive index detector (RI-101, Perkin Elmer, Shelton, CT, USA). An acetonitrile/water mixture (65:35, v/v) was employed as the mobile phase, as previously reported (Verni et al. 2019). Prior to analysis, freeze-dried samples were reconstituted in 1 mL of acetonitrile (65%, v/v). Sugar identification and calibration curves were obtained using analytical standards of lactose, glucose, and galactose (Sigma-Aldrich, Milan, Italy).

Total free amino acid (TFAA) content was determined using a Biochrom 30 + series amino acid analyzer (Biochrom Ltd., Cambridge Science Park, UK) equipped with a lithium cation-exchange column (0.46 cm internal diameter) and post-column ninhydrin derivatization, following the method described by Verni et al. (2021). Fat and ash contents were quantified according to international standard procedures, namely the Gerber method (2008) and AOAC method 945.4 (2016), respectively. The carbon-to-nitrogen (C/N) ratio of the substrates was determined using a CN802 elemental analyzer (VELP Scientifica, Usmate Velate, Italy).

The viable cell density of lactic acid bacteria in RCEW was assessed by plate counting on De Man, Rogosa and Sharpe (MRS) agar (Oxoid, Basingstoke, Hampshire, UK) supplemented with cycloheximide (0.1 g L⁻^1^). Plates were incubated at 30 °C for 48 h under anaerobic conditions using AnaeroGen sachets and AnaeroJar systems (Oxoid). Yeasts were enumerated on Yeast Peptone Dextrose Agar (YPDA; Sigma-Merck, Darmstadt, Germany) supplemented with chloramphenicol (0.1 g L⁻^1^), following incubation at 30 °C for 72 h. Total mesophilic aerobic bacteria were quantified on Plate Count Agar (PCA; Oxoid) after incubation at 30 °C for 48 h, while Enterobacteriaceae were determined using Violet Red Bile Glucose Agar (VRBGA; Oxoid) incubated at 37 °C for 24 h. All microbiological analyses were conducted in triplicate.

The characterization of RCEW-derived substrates (pRCEW, sRCEW, and mRCEW; described below) was carried out following the same analytical procedures applied to the native substrate.

### Preparation, pretreatment, and supplementation of cultivation substrates

#### Ricotta cheese exhausted whey–based substrate (mRCEW)

According to Longo et al. (2025), a mixture (mRCEW) of pasteurized and sterilized ricotta cheese exhausted whey at a 75:25 ratio was used as the main cultivation substrate. Thermal treatments and the mixing ratio were selected based on previous work (Longo et al. 2025), balancing microbial growth and PHA production performance with process efficiency and data comparability across PHA-accumulating microorganisms. In addition, the use of differently treated RCEW fractions allowed modulation of the substrate nitrogen content and carbon-to-nitrogen (C/N) ratio, two key parameters affecting biomass formation and PHB accumulation.

In particular, RCEW was partially pasteurized at 63 °C for 30 min (pRCEW) and partially sterilized at 121 °C for 30 min under a pressure of 15 psi above atmospheric pressure (sRCEW). Pasteurization was applied to reduce the microbial load while largely preserving the native nutritional composition of the substrate, whereas sterilization promoted extensive whey protein denaturation and precipitation, thereby reducing nitrogen availability. Following thermal treatment, pRCEW and sRCEW were centrifuged at 12,000 × g for 15 min at 4 °C, and the resulting supernatants were combined to prepare the final substrate mRCEW, while the pellets, mainly consisting of denatured whey proteins, were discarded. The final 75:25 pRCEW:sRCEW ratio was selected to provide moderate nitrogen limitation while maintaining sufficient nutrient availability to sustain microbial growth, as previously demonstrated by Longo et al. (2025). mRCEW was supplemented with 0.1% (v/v) trace solution (TS) and 10% mineral medium (MM), as previously proposed by Zafar et al. (2012); Sharma et al. (2017) and further modified by Longo et al. (2025) to adapt the formulation to the mineral composition of RCEW. In detail, TS had the following composition: FeSO_4_ × 7H_2_O (20 g L^−1^); H_3_BO_4_ (0,3 g L^−1^); CoCl_2_ × 6H_2_O (0,20 g L^−1^); ZnSO_4_ × 7H_2_O, 0,03 g L^−1^; MnCl_2_ × 4H_2_O, 0.03 g L^−1^; (NH_4_)_6_Mo_7_O_24_ × 4H_2_O, 0,03 g L^−1^; NiSO_4_ × 7H_2_O, 0,03 g L^−1^; CuSO_4_ × 5H_2_O, 0,01 g L^−1^. MM had the following composition: Na_2_HPO_4_, 9 g L^−1^; KH_2_PO_4_, 4,0 g L^−1^; (NH_4_)_2_SO_4_, 3,0 g L^−1^; MgSO_4_ × 7H_2_O, 0.30 g L^−1^; citric acid, 0.10 g L^−1^; yeast extract, 0.15 g L^−1^; CaCl_2_, 0.01 g L^−1^.

A commercial β-galactosidase from *Kluyveromyces lactis* Lactozym Pure 6500 L (1320 U/mL) (Novozymes, Bagsvaerd, Denmark) was added to mRCEW at a concentration of 0.1% (v/v) (Rodriguez-Colinas et al. 2014). According to previous experiments (Longo et al. 2025), more than 90% of lactose hydrolysis was achieved within the first 6 h of incubation.

#### Coffee oil extraction and mRCEW supplementation

Waste ground coffee powder originating from the coffee roasting industry was used in its raw form (without prior coffee extraction) to obtain a coffee oil extract (EOC) employed as an mRCEW supplement. The coffee powder exhibited a moisture content of 4.1% and the following chemical composition on a dry weight basis: proteins, 10.4 g/100 g; lipids, 15.4 g/100 g; and carbohydrates, 28.5 g/100 g.

Powdered coffee waste was weighed into a cellulose thimble (3.5 cm in diameter and 9.5 cm in height) and placed in the central glass chamber of a Soxhlet extractor. Coffee oil was extracted using 300 mL of n-hexane, and the extraction process was conducted for approximately 4 h (Hanif et al. 2019; Ahangari and Sargolzaei 2013). After completion of the extraction, the solvent was removed by vacuum distillation using a rotary evaporator (RotoVap 5L RE-501, Vevor, Germany), recovered and further reused in the following extraction cycle.

Following solvent removal, the recovered coffee oil extract (EOC) was directly supplemented to mRCEW before inoculation at concentrations of 0.5, 1.0, and 1.5% (v/v), according to the literature (Bhatia et al. 2018). Cultures without EOC supplementation were used as controls. The supplemented media were then inoculated with *C. necator* DSM 428 and incubated under the conditions described above.

### Induced oxidative stress conditions

The production of PHA under oxidative stress conditions was investigated in mRCEW by applying controlled additions of ethanol or hydrogen peroxide during cultivation. Ethanol (96%, v/v) and hydrogen peroxide (3%, v/v) were supplemented at 24 or 60 h of cultivation, at final concentrations of 0.50% or 0.25% (w/w) for ethanol and 5.0 or 2.5 mmol L⁻^1^ for hydrogen peroxide, respectively. These conditions were selected to evaluate the effect of both the timing and intensity of oxidative stress induction on microbial growth and PHA accumulation. Fermentations were conducted for a total duration of 120 h to capture potential delayed responses to stress, following an approach previously described by Obruca et al. (2010a, b).

### Bacterial growth and PHA production

The ability of *C. necator* DSM 428 to synthesize PHA was evaluated in mRCEW, mRCEW + EOC, and mRCEW under oxidative stress conditions. Cultivation experiments were carried out at 30 °C in 200 mL working volume batches placed in 0.5 L Pyrex glass Erlenmeyer flasks under agitation at 180 rpm. Based on previous studies (Zafar et al. 2012), the fermentation duration was set to 72 h. The culture pH was adjusted to 7.0 at the beginning of the experiment (t₀) using 1 M NaOH and subsequently corrected every 3 h.

To prevent yeast contamination, cycloheximide was added to all cultivations, including controls, at a final concentration of 0.10 g L⁻^1^. The inoculum of *C. necator* DSM 428 was prepared by adding 10% (v/v) of an overnight-grown culture. Lactose, glucose and galactose during fermentation were monitored as described above.

### Bioplastic production

Microbial biomass, expressed as dry cell weight (DCW), as well as the amount of purified PHA accumulated under the different experimental conditions, was quantified following previously reported methodologies (Raho et al. 2020), with specific modifications as described elsewhere (Montemurro et al. 2022).

#### Dry cell weight

Following cultivation, biomass was recovered by separating the spent medium from the cells via centrifugation at 12,000 × g for 50 min at 4 °C. To minimize cell disruption, the harvested biomass was washed with a 0.9% (w/v) NaCl solution and centrifuged once more under the same conditions. The resulting pellet was dried at 60 °C for at least 24 h and gravimetrically determined as dry cell weight (DCW).

#### Quantification of intracellular polyhydroxyalkanoates (PHA)

Polyhydroxyalkanoates (PHA) were recovered from dry cell biomass by chemical digestion using a biphasic system composed of sodium hypochlorite and chloroform (CHCl_3_). Specifically, dried biomass was treated with a mixture of 12% (v/v) sodium hypochlorite and chloroform at a ratio of 12.5 mL of each reagent per gram of dry cells. The suspension was incubated in a water bath at 30 °C for 90 min, with periodic vortexing to ensure complete polymer solubilization. After phase separation, the organic fraction containing PHA was collected and further clarified by centrifugation at 12,800 × g for 10 min at 4 °C to remove residual impurities. Following evaporation of the organic solvent, the resulting solid fraction, corresponding to raw PHA, was recovered and gravimetrically determined.

The intracellular PHA content, defined as the fraction of PHA relative to the total DCW, was calculated according to the following equation (Eq. 1):

1 $$\%PHA/DCW\hspace{0.17em}=\hspace{0.17em}\lbrack PHA\;(g\;L^{-1})/DCW\;(g\;L^{-1})\rbrack\hspace{0.17em}\times\hspace{0.17em}100$$

Crude PHA was solubilized in chloroform, and polymer purification was achieved by adding cold ethanol at a volumetric ratio of 1:10 (v/v), inducing polymer precipitation. The resulting purified PHA, corresponding to the solid fraction, was recovered by centrifugation at 12,800 × g for 10 min at 4 °C and subsequently subjected to further characterization.

### Evaluation of PHB production under controlled bioreactor conditions

#### Batch and fed-batch cultivation of *C. necator* DSM 428 in stirred-tank bioreactor

Biopolymer production by *Cupriavidus necator* DSM 428 was evaluated in a 2-L stirred-tank bioreactor (Lambda Minifor, Lambda CZ, Brno, Czech Republic). Cultivations were carried out using mRCEW supplemented with trace solution (TS) and mineral medium (MM). Batch runs were performed with a working volume of 1.2 L under automatic control of pH and temperature, maintained at 7.20 ± 0.01 and 30.0 ± 0.1 °C, respectively, for a total cultivation time of 72 h. In particular, the pH was automatically controlled by the addition of sterile 2 M NaOH and 2 M HCl solutions through the bioreactor control system. Agitation was set at 200 rpm, and sterile filtered air was supplied at a flow rate of 1.0 vvm (vessel volumes of air per minute per volume of culture medium). No antifoam agent was used.

Two cultivation strategies were investigated: batch fermentation (b/mRCEW) and fed-batch cultivation (fb/mRCEW). In both cases, the initial working volume was 1.0 L. For fed-batch experiments, a single feeding step was carried out at 24 h by adding 50% of the initial working volume (0.5 L) of sRCEW supplemented with β-galactosidase. This feeding strategy was intentionally designed to maintain nitrogen-limited conditions while replenishing carbon availability, thereby promoting continued PHB accumulation rather than biomass formation. Additional nitrogen supplementation was deliberately avoided, as excessive nitrogen availability is known to redirect carbon flux toward cell growth at the expense of storage polymer synthesis. Indeed, the fed substrate exhibited a substantially lower protein content compared to mRCEW (12 mg L⁻^1^ vs. 330 mg L⁻^1^), allowing an increase in carbon availability while limiting additional nitrogen input.

In both batch and fed-batch cultivations, oxidative stress was induced at 24 h of fermentation by the addition of hydrogen peroxide at a final concentration of 5.0 mmol L⁻^1^.

#### Chlorine-free extraction and recovery of PHB

An alternative chlorine-free extraction method was applied for PHB recovery, following the protocol proposed by Salvatori et al. (2023). Briefly, harvested biomass was centrifuged, washed with 0.9% (w/v) NaCl solution, dried at 60 °C for 24 h, and weighed to determine cell dry matter. The dried biomass was subjected to alkaline hydrolysis using a 0.2 M NaOH solution for 4 h under agitation, at 25 °C, followed by centrifugation and washing steps. The resulting pellet was then treated with a 1.5% (w/v) hydrogen peroxide solution for 1 h to complete the removal of non-PHA cellular material. After final washing and drying at 60 °C for 24 h, the recovered solid fraction was collected and weighed as raw PHB. This chlorine-free treatment was adopted as a sustainable alternative to conventional extraction methods (Pagliano et al. 2021).

### PHA characterization

#### Composition by gas chromatography (GC) analysis

The monomer composition of the extracted polymer was determined by gas chromatography (GC) through injection of 1 μL of the organic phase into an Agilent 8860 GC system fitted with an HP-5 capillary column (30 m × 320 μm × 0.25 μm). Nitrogen was employed as the carrier gas at a flow rate of 10 mL/min. The oven temperature program started at 100 °C and was held for 1 min, then ramped to 130 °C at 12 °C/min, followed by a further increase to 250 °C at 25 °C/min. The flame ionization detector (FID) temperature was set at 300 °C. Sample preparation for PHA analysis was performed according to previously reported procedures (Braunegg et al. 1978).

#### Average molecular weight and polydispersity index

The average molecular weight (Mw) and polydispersity index (PDI = Mw/Mn, with Mn being the number average molecular weight) of the extracted PHA were assessed by gel permeation chromatography (GPC). The system consisted of a JASCO PU-4180 pump, a guard column, two columns connected in series (TSKgel G6000-HHR and TSKGel GMHHR-H), a column oven (JASCO CO-4060), and a refractive index detector (JASCO RI-4030), following the procedure previously described by Salvatori et al. (2023).

### Statistical analysis

All biochemical measurements were performed using at least three independent biological replicates, with each replicate analyzed in duplicate. The resulting data were evaluated by one-way analysis of variance (ANOVA), and differences among treatment means were assessed using Tukey’s post hoc test at a significance level of *p* < 0.05, employing IBM SPSS Statistics version 26 (IBM Corporation, New York City, NY, USA).

## Results

### RCEW and RCEW-derived substrates characterization

The chemical and microbiological characterization of ricotta cheese exhausted whey (RCEW) and of the derived substrates obtained by thermal treatments and mixing is reported in Table 1. RCEW showed an acidic pH (5.2), high lactose content (3.81% w/v), and low concentrations of proteins and lipids, confirming its suitability as a carbon-rich and nitrogen-poor substrate. Thermal processing resulted in marked compositional changes, particularly affecting the nitrogenous fraction. Pasteurization (followed by centrifugation and pellet removal) led to a moderate reduction in protein content, whereas sterilization caused an almost complete removal of proteins, with a corresponding decrease in total nitrogen. As a consequence, the C/N ratio increased from 27 in RCEW to 34 in pRCEW and reached extremely high values in sRCEW (C/N ≈ 1000). The mixed substrate (mRCEW), obtained by combining pRCEW and sRCEW at a 75:25 ratio, displayed intermediate characteristics, with a C/N ratio of 44.

**Table 1:** Chemical and microbiological characterization of RCEW and derived substrates

Parameter	RCEW	pRCEW	sRCEW	mRCEW
pH	5.2 ± 0.1^a^	5.3 ± 0.1^a^	5.4 ± 0.1^a^	5.3 ± 0.1^a^
Total nitrogen (% w/v)	0.060 ± 0.010^a^	0.052 ± 0.005^a^	0.002 ± 0.002^c^	0.039 ± 0.003^b^
Proteins (% w/v)	0.38 ± 0.06^a^	0.33 ± 0.03^a^	0.01 ± 0.01^c^	0.25 ± 0.02^b^
Total free amino acids (mg L^−1^)	403 ± 25^a^	348 ± 12^b^	256 ± 18^c^	325 ± 21^b^
Lactose (% w/v)	3.81 ± 0.15^a^	3.80 ± 0.10^a^	3.78 ± 0.03^a^	3.79 ± 0.10^a^
Glucose (% w/v)	< 0.01	< 0.01	< 0.01	< 0.01
Galactose (% w/v)	< 0.01	< 0.01	< 0.01	< 0.01
Fat (% w/v)	0.20 ± 0.03^a^	0.17 ± 0.01^a^	0.05 ± 0.02^c^	0.14 ± 0.02^b^
Ash (% w/v)	1.20 ± 0.05^a^	1.18 ± 0.05^a^	1.05 ± 0.05^a^	1.15 ± 0.05^a^
C/N ratio	27^d^	34^c^	1019^a^	44^b^
Total aerobic bacteria (log CFU/mL)	5.30 ± 0.11^a^	3.00 ± 0.15^b^	nd	2.88 ± 0.09^b^
Lactic acid bacteria (log CFU/mL)	4.56 ± 0.21^a^	2.26 ± 0.10^b^	nd	2.14 ± 0.10^b^
Yeasts (log CFU/mL)	3.21 ± 0.20^a^	< 1^b^	nd	< 1^b^
Enterobacteriaceae (log CFU/mL)	< 1	nd	nd	nd

^a–c^Values in the same row with different superscript letters are significantly different at *P* < 0.05

Lactose concentration remained essentially unchanged across all substrates, indicating that thermal treatments did not affect carbohydrate availability. Lipid content slightly but progressively decreased from RCEW to pRCEW and sRCEW, while ash content showed no significant differences (*P* > 0.05), suggesting limited mineral loss during processing. Microbiological analyses highlighted a substantial reduction in viable microbial populations following thermal treatments: pasteurization significantly decreased total aerobic bacteria, lactic acid bacteria, and yeasts, whereas sterilization resulted in the complete absence of detectable microorganisms. Accordingly, mRCEW exhibited low residual microbial loads, reflecting the combined effect of pasteurized and sterilized fractions.

### PHB production in shake-flask cultures and effect of coffee oil supplementation

The growth performance and PHB production of *C. necator* DSM 428 cultivated in mRCEW with or without supplementation of coffee oil extract (EOC) are reported in Table 2. In the absence of EOC, the strain reached a dry cell weight of 1.60 ± 0.04 g L⁻^1^ after 72 h of cultivation and accumulated 1.14 ± 0.03 g L⁻^1^ of PHB, corresponding to 71.4 ± 2.4% of the cell dry matter. These results confirm the suitability of mRCEW as a carbon-rich substrate able to support both growth and intracellular PHB accumulation of the strain.

**Table 2:** Biomass formation and PHB accumulation by *Cupriavidus necator* DSM 428 in mRCEW with different levels of coffee oil extract (EOC) supplementation

Fermentation substrate	Dry Cell Weight g L^−1^	PHB g L^−1^	% PHB/DCW
mRCEW	1.60 ± 0.04^d^	1.14 ± 0.03^c^	71.4 ± 2.4^a^
*Supplementation with EOC*			
mRCEW + 0.5% EOC	4.41 ± 0.04^c^	2.86 ± 0.01^a^	65.0 ± 0.8^b^
mRCEW + 1.0% EOC	4.69 ± 0.02^a^	2.81 ± 0.02^a^	59.8 ± 0.6^c^
mRCEW + 1.5% EOC	4.56 ± 0.02^b^	2.26 ± 0.02^b^	49.7 ± 0.6^d^

^a–d^Values in the same column with different superscript letters are significantly different at *P* < 0.05

Supplementation of mRCEW with EOC markedly enhanced biomass formation and absolute PHB production. The addition of 0.5% (v/v) EOC resulted in a nearly threefold increase in biomass concentration, reaching 4.41 ± 0.04 g L⁻^1^, accompanied by a PHB concentration of 2.87 ± 0.01 g L⁻^1^. Despite the substantial increase in polymer titer, the intracellular PHB fraction decreased to 65.0 ± 0.8 DCW, indicating that EOC primarily stimulated cell growth rather than selectively promoting polymer accumulation.

A similar trend was observed at 1.0% (v/v) EOC, where biomass formation further increased to 4.69 ± 0.02 g L⁻^1^, while PHB concentration remained comparable (2.81 ± 0.02 g L⁻^1^). Under these conditions, the intracellular PHB content declined to 59.8 ± 0.6% DCW, suggesting a progressive shift toward biomass formation as EOC concentration increased.

At the highest supplementation level tested (1.5% v/v EOC), biomass concentration remained high (4.56 ± 0.02 g L⁻^1^), but PHB production decreased to 2.26 ± 0.02 g L⁻^1^, with a corresponding intracellular PHB content of 49.7 ± 0.6% DCW. This reduction indicates that excessive EOC supplementation negatively affected polymer accumulation, possibly due to metabolic imbalance or inhibitory effects associated with high lipid availability.

Overall, EOC supplementation exerted a strong positive effect on biomass formation and absolute PHB production in shake-flask cultures, while simultaneously reducing the intracellular PHB fraction at increasing concentrations. The most favorable balance between biomass growth and PHB accumulation was observed at 0.5% (v/v) EOC, which yielded the highest PHB titer while maintaining a relatively high polymer content within the cells.

### Effect of oxidative stress induction on biomass formation and PHB accumulation

To assess the prolonged effects of oxidative stress on microbial growth and PHB accumulation, in addition to short-term responses, cultures were monitored up to 120 h of cultivation. This extended observation period was selected to account for potential delayed responses to stress induction, particularly with respect to intracellular polymer accumulation under prolonged nutrient limitation.

The effect of oxidative stress on biomass formation and PHB accumulation by *C. necator* DSM 428 cultivated in mRCEW was evaluated by supplementing hydrogen peroxide or ethanol at different cultivation times and concentrations (Table 3). Control cultures grown in mRCEW without stress induction showed a progressive decline in PHB content between 72 and 120 h (accompanied by a slight reduction in biomass weight), with PHB accounting for approximately 64.3% of the cell dry matter at the end of cultivation.

**Table 3:** Biomass formation and PHB accumulation by *C. necator* DSM 428 under oxidative stress conditions induced by hydrogen peroxide or ethanol

Fermentation substrate	72 h			120 h		
	Dry cell Weight g L^−1^	PHB g L^−1^	% PHB/DCW	Dry Cell Weight g L^−1^	PHB g L^−1^	% PHB/DCW
mRCEW (control)	1.60 ± 0.04^d^	1.14 ± 0.03^b^	71.4 ± 2.4^a^	1.34 ± 0.01^c^	0.86 ± 0.01^c^	64.3 ± 1.2^b^
*Oxidative stress induction*						
5.0 mmol L⁻^1^ H_2_O_2_ added at 24 h	2.80 ± 0.02^a^	1.71 ± 0.01^a^	61.2 ± 0.7^b^	1.78 ± 0.01^a^	1.44 ± 0.02^a^	80.8 ± 1.8^a^
5.0 mmol L⁻^1^ H_2_O_2_ added at 60 h	1.34 ± 0.02^d^	0.52 ± 0.00^e^	39.2 ± 0.8^d^	1.29 ± 0.01^c^	0.52 ± 0.01^d^	40.8 ± 1.3^d^
2.5 mmol L⁻^1^ H_2_O_2_ added at 60 h	1.77 ± 0.02^c^	0.65 ± 0.01^d^	36.6 ± 0.5^e^	1.46 ± 0.02^b^	0.72 ± 0.01^c^	49.6 ± 0.8^c^
0.50% (w/w) Ethanol added at 24 h	2.42 ± 0.02^b^	0.89 ± 0.01^c^	36.7 ± 0.6^e^	1.74 ± 0.01^a^	1.02 ± 0.02^b^	58.9 ± 1.0^c^
0.50% (w/w) Ethanol added at 60 h	1.30 ± 0.02d	0.67 ± 0.01^d^	51.9 ± 1.2^c^	1.21 ± 0.01^d^	0.49 ± 0.00^d^	40.2 ± 0.6^d^
0.25% (w/w) Ethanol added at 60 h	1.89 ± 0.01^c^	0.59 ± 0.01^e^	31.1 ± 0.7^e^	1.14 ± 0.02^d^	0.39 ± 0.00^e^	34.1 ± 0.7^e^

^a–e^Values in the same column with different superscript letters are significantly different at *P* < 0.05

**Fig. 2:**
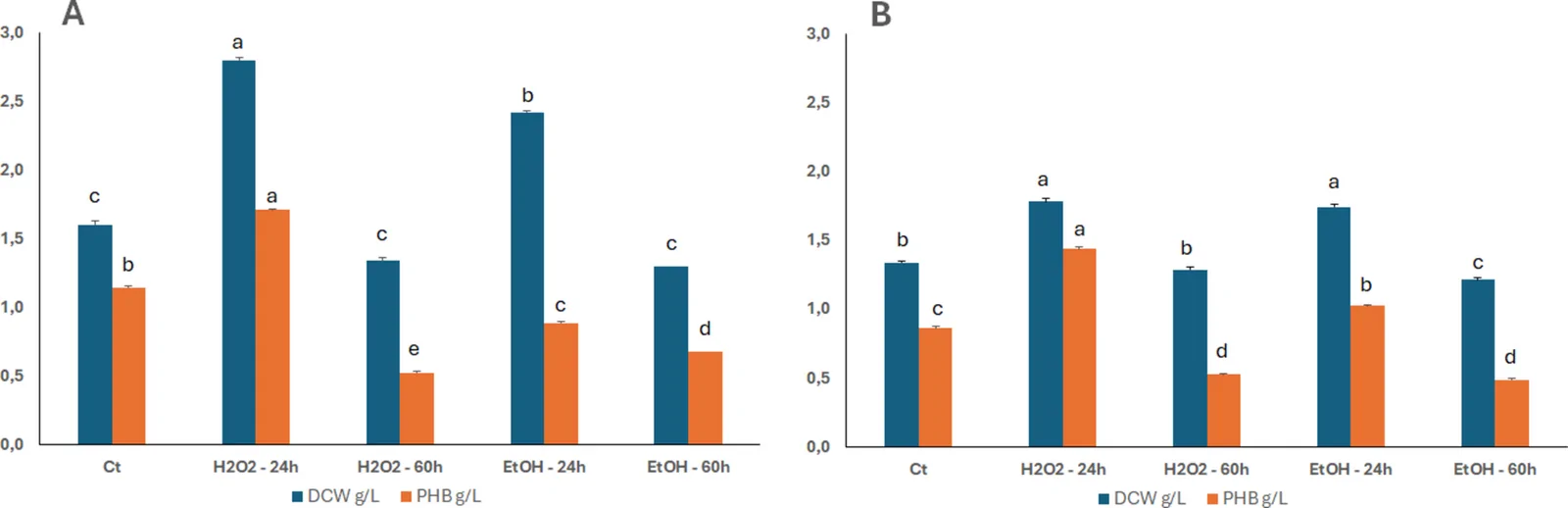
Effect of oxidative stress timing on biomass formation (dry cell weight, DCW) and PHB accumulation (g L⁻.^1^) by *Cupriavidus necator* DSM 428 cultivated in mRCEW after 72 h (**A**) and 120 h (**B**) of cultivation following oxidative stress induction with hydrogen peroxide or ethanol (added at 24 or 60 h). Data are reported as means of independent biological replicates ± standard deviation. Within each panel, different letters indicate statistically significant differences in DCW or PHB values (*P* < 0.05)

The response of *C. necator* to hydrogen peroxide strongly depended on both the timing and concentration of stress induction. When hydrogen peroxide was added at 24 h of cultivation, biomass formation at 72 h increased compared to the unstressed control, reaching approximately 2.8 g L⁻^1^. Under these conditions, PHB accumulation at 72 h accounted for about 61% of the cell dry matter. Upon extending cultivation to 120 h, a marked increase in intracellular PHB accumulation was observed, with PHB representing approximately 81% of the cell dry matter, despite a moderate reduction in biomass concentration (Fig. 2 and Table 3).

In contrast, hydrogen peroxide supplementation at 60 h resulted in substantially lower biomass formation and PHB accumulation. In these cultures, intracellular PHB contents remained below 41% of the cell dry matter at both 72 and 120 h. Reducing the hydrogen peroxide concentration by half partially alleviated this effect, leading to moderate increases in biomass and PHB accumulation; however, intracellular PHB levels remained markedly lower than those obtained with early stress induction (Fig. 2 and Table 3).

Ethanol supplementation also influenced biomass formation and PHB accumulation in a time- and concentration-dependent manner. When ethanol was added at 24 h, biomass formation at 72 h increased relative to the unstressed control, whereas intracellular PHB content was reduced to approximately 37% of the cell dry matter. Prolonged cultivation to 120 h resulted in a partial recovery of PHB accumulation, with PHB representing nearly 59% of the cell dry matter.

Conversely, ethanol addition at 60 h led to less favorable outcomes. Both biomass formation and PHB accumulation were lower than those observed with early supplementation, and PHB content further decreased during extended cultivation. Similarly, halving the ethanol concentration at 60 h did not result in significant improvements, with intracellular PHB contents remaining below 35% of the cell dry matter at both sampling times (Fig. 2 and Table 3).

Overall, oxidative stress induction markedly affected PHB production by *C. necator* DSM 428, with hydrogen peroxide added at early cultivation stages promoting high and sustained intracellular polymer accumulation during prolonged cultivation, whereas ethanol supplementation resulted in more limited and timing-dependent effects.

### PHB production in bioreactor: batch and fed-batch cultivation under controlled conditions

The performance of *Cupriavidus necator* DSM 428 cultivated in mRCEW was further evaluated under controlled conditions in a stirred-tank bioreactor, in order to assess process efficiency and to compare batch and fed-batch strategies (Table 4). Bioreactor experiments were conducted under controlled pH, temperature, aeration, and agitation, and oxidative stress was induced by hydrogen peroxide addition at 24 h, based on the results obtained in shake-flask cultivations.

**Table 4:** Biomass formation and PHB accumulation by *C. necator* DSM 428 cultivated in bioreactor under batch and fed-batch conditions with hydrogen peroxide (5.0 mmol L⁻^1^) induction at 24 h, as determined by conventional and chlorine-free extraction methods

Fermentation substrate	Dry cell weight g L^−1^	*Conventional extraction*		*Chlorine-free recovery*	
		PHB g L^−1^	% PHB/DCW	PHB g L^−1^	% PHB/DCW
b/mRCEW	3.96 ± 0.12^b^	2.75 ± 0.05^b^	69.4 ± 0.2^a^	3.08 ± 0.06^b^	77.8 ± 0.8^a^
Fb/mRCEW	4.94 ± 0.55^a^	3.56 ± 0.12^a^	72.2 ± 0.2^a^	3.85 ± 0.12^a^	77.9 ± 1.2^a^

^a–b^Values in the same column with different superscript letters are significantly different at *P* < 0.05

Compared to shake-flask experiments, bioreactor batch cultivation resulted in enhanced biomass formation and PHB production. After 72 h of cultivation, batch bioreactor cultures reached a dry cell weight of approximately 4.0 g L⁻^1^ and accumulated 2.75 g L⁻^1^ of PHB, corresponding to nearly 70% of the cell dry matter. Implementation of a fed-batch strategy further improved process performance. The addition of sRCEW supplemented with β-galactosidase at 24 h led to increased substrate availability without significantly increasing nitrogen input, resulting in higher final biomass and PHB concentrations. Under fed-batch conditions, *C. necator* reached a dry cell weight of approximately 4.9 g L⁻^1^ and accumulated 3.56 g L⁻^1^ of PHB, corresponding to more than 72% of the cell dry matter.

In particular, the PHB average volumetric productivity under batch bioreactor conditions corresponded to 0.038 g L⁻^1^ h⁻^1^, whereas fed-batch cultivation increased PHB productivity to 0.049 g L⁻^1^ h⁻^1^.

**Fig. 3:**
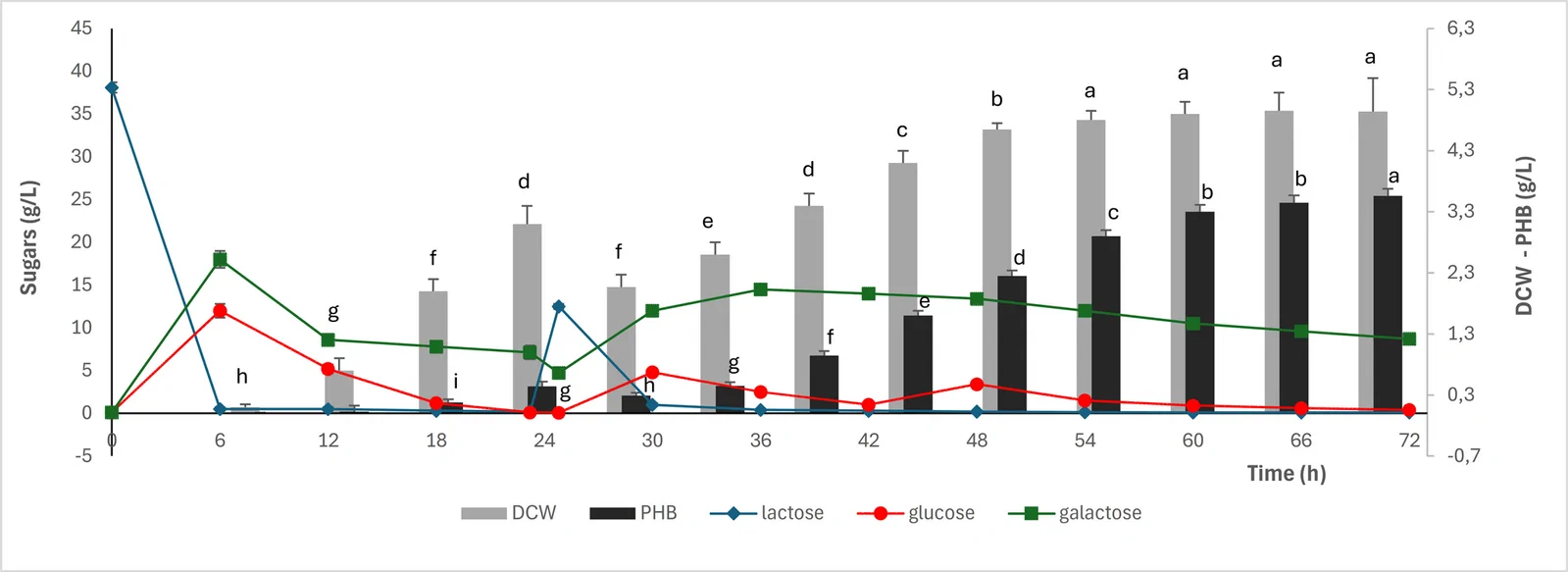
Time-course of sugar consumption and DCW/PHB accumulation during fed-batch cultivation of *C. necator* DSM 428 in mRCEW under bioreactor conditions. Data are the means of three independent replicates. ^a−i^DCW and PHB values with different letters, differ significantly (*P* < 0.05)

The evolution of sugars and PHB accumulation during fed-batch cultivation is detailed in Fig. 3 (and supplementary Table S1). In both the initial batch phase and following the feeding step, lactose was rapidly hydrolyzed following β-galactosidase addition, with more than 90% conversion observed within the first hours after each supplementation. The resulting monosaccharides were consumed sequentially, with glucose being preferentially and more rapidly assimilated than galactose. This consumption pattern was consistent throughout the cultivation and was particularly evident after the feeding step, where glucose depletion preceded galactose utilization.

Overall, the fed-batch strategy allowed prolonged carbon availability while maintaining nitrogen-limited conditions. This operational regime favored intracellular PHB accumulation while still permitting biomass growth throughout the cultivation period. Compared to batch operation, fed-batch cultivation resulted in higher final biomass and PHB concentrations, demonstrating the beneficial effect of carbon feeding on overall process productivity under the experimental conditions adopted.

The performance of a chlorine-free extraction protocol was compared with that of the conventional chloroform-based method for PHB recovery from bioreactor cultivations (Table 4). In batch cultures grown on mRCEW (b/mRCEW), conventional extraction resulted in a PHB concentration of 2.75 g L⁻^1^, corresponding to 69.4% of the cell dry matter. When the chlorine-free protocol was applied to the same biomass, PHB recovery increased to 3.08 g L⁻^1^, corresponding to 77.8% of the cell dry matter.

**Fig. 4:**
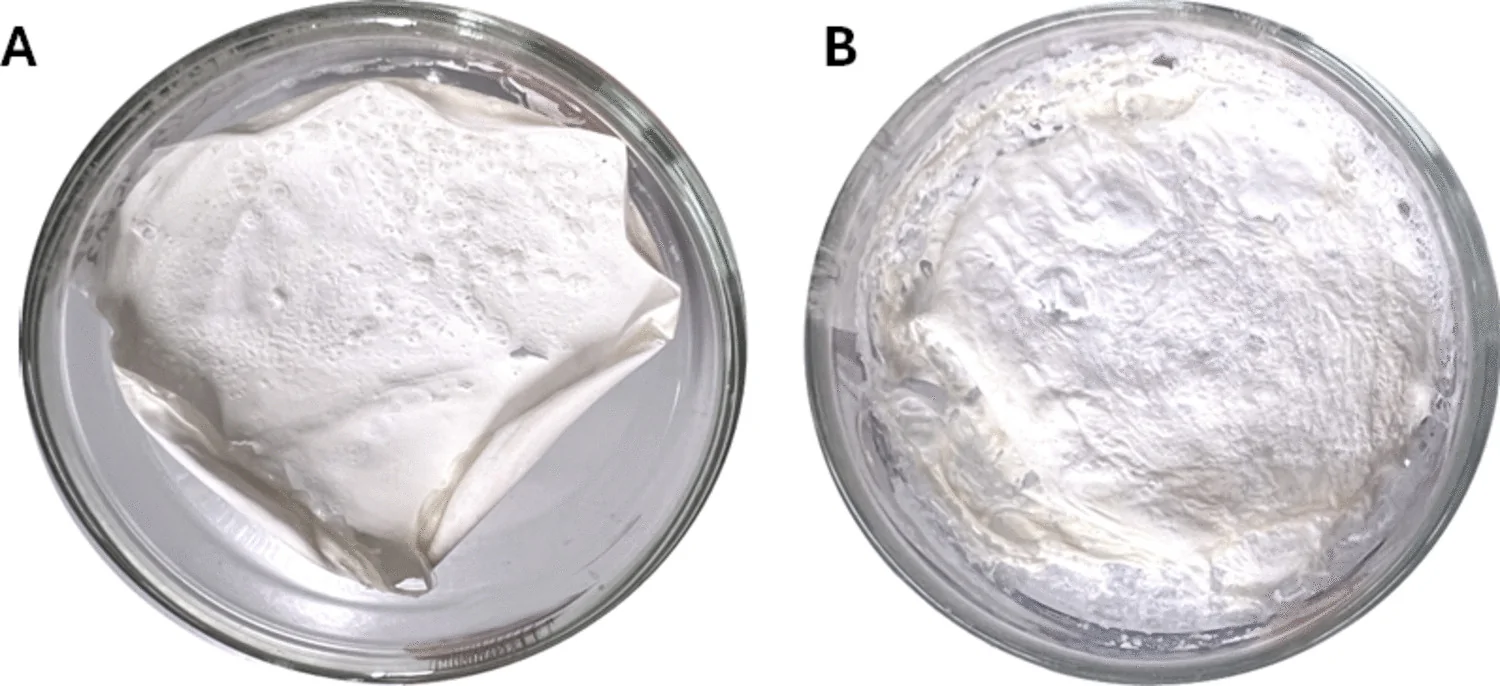
Macroscopic appearance of PHB recovered by conventional (**A**) and chlorine-free (**B**) extraction methods

A similar trend was observed under fed-batch conditions (fb/mRCEW). Using the conventional extraction method, PHB accumulation reached 3.56 g L⁻^1^, accounting for 72.2% of the dry cell weight. In contrast, application of the chlorine-free protocol resulted in a PHB concentration of 3.85 g L⁻^1^ and an intracellular PHB content of 77.9% of the cell dry matter. Overall, the chlorine-free extraction method yielded PHB recovery values comparable to, or slightly higher than, those obtained with the conventional solvent-based approach, while avoiding the use of chlorinated organic solvents (Fig. 4).

PHB recovered from fed-batch bioreactor cultivation of *C. necator* DSM 428 under oxidative stress induction at 24 h and obtained through chlorine-free downstream recovery was subjected to physicochemical characterization to assess polymer composition and molecular weight distribution. Gas chromatography analysis revealed that the recovered polymer consisted exclusively of 3-hydroxybutyrate units, with a 3HB content of 100% (w/w), indicating the production of a PHB homopolymer.

Molecular weight analysis showed a weight-average molecular weight (Mw) of 876 kDa, with a polydispersity index (PDI) of 1.9, reflecting a moderately broad molecular weight distribution typical of PHB produced from complex waste-derived substrates under controlled fed-batch cultivation conditions.

## Discussion

The valorization of cheese whey (CW) and ricotta cheese exhausted whey (RCEW) remains a critical issue for the dairy sector, particularly in countries such as Italy where production is geographically fragmented and largely driven by small- to medium-sized enterprises. As extensively reported, approximately 9–10 L of whey are generated per kilogram of cheese, and the subsequent production of whey cheeses further amplifies the volume of liquid by-products, with up to 18 L of RCEW produced per kilogram of ricotta-type cheese (Pires et al. 2021; Raho et al. 2020). Despite its residual nutritional value (mainly lactose, minerals, and soluble nitrogen), RCEW is still frequently underutilized or diverted to low-value applications, making it an attractive but technically challenging substrate for microbial bioprocesses aimed at producing value-added compounds such as bioplastics.

Within this framework, *Cupriavidus necator* has long been recognized as one of the most robust and well-characterized microbial platforms for polyhydroxyalkanoate (PHA) production. Its capacity to accumulate high intracellular fractions of poly(3-hydroxybutyrate) (PHB), together with its metabolic flexibility and extensive physiological characterization, has made it a reference organism in both fundamental and applied biopolymer research (Morlino et al. 2023; Weldon and Euler 2025). While genetic engineering has been widely explored to expand substrate utilization or improve polymer yields, such approaches may face regulatory constraints in several European contexts and can complicate process transfer to industrial settings. For this reason, the present study deliberately focused on a non-recombinant strain (DSM 428), adopting a strategy centered on substrate and process optimization rather than microbial engineering, with the explicit goal of developing an operationally simple process based on agro-industrial side-stream valorization.

A key element of this strategy was the conditioning of RCEW through simple thermal treatments (pasteurization and sterilization), which were exploited not only for hygienic control but also as a tool to modulate substrate composition. As demonstrated by Longo et al. (2025), thermal processing of RCEW promotes whey protein denaturation and partial removal, effectively reducing the organic nitrogen fraction and shifting the carbon-to-nitrogen (C/N) ratio without the need for extensive fractionation or complex downstream handling. In that study, a mixed stream obtained from pasteurized and sterilized RCEW (RCEW-III) reached a C/N ratio of approximately 44 and supported PHA accumulation in *Azohydromonas lata*. Although C/N ratios in the range of 20–30 have frequently been reported as favorable for PHA production in defined media, the optimal value is highly dependent on strain, substrate composition, and nitrogen bioavailability. Previous studies performed on whey-derived substrates have also identified favorable PHA production at substantially higher nominal C/N ratios, particularly when nitrogen is predominantly associated with proteins and peptide fractions (Longo et al. 2025). In our system, mRCEW (C/N = 44) provided the best balance between growth and PHB accumulation, whereas both lower (C/N = 34) and much higher (C/N = 1019) ratios resulted in inferior overall performances. These findings suggest that nitrogen accessibility rather than the nominal C/N ratio alone is the main driver of PHB accumulation under the tested conditions. The mRCEW formulation adopted here follows the same conceptual approach: by combining thermal conditioning with limited mineral and trace supplementation, a C/N regime compatible with PHB biosynthesis in *C. necator* was achieved without constructing a fully synthetic medium.

Carbon accessibility represents another critical aspect when working with RCEW. Native *C. necator* strains are unable to directly metabolize lactose, and several studies have addressed this limitation either by genetic modification or by using lactose-free whey derivatives (Morlino et al. 2023). In the present work, lactose utilization was enabled through enzymatic hydrolysis via β-galactosidase, a strategy previously demonstrated as effective in RCEW-based PHA processes (Raho et al. 2020; Longo et al. 2025). In both batch and fed-batch cultivations, lactose was rapidly hydrolyzed following enzyme addition, leading to the release of glucose and galactose. Consistent with established knowledge of *C. necator* physiology, glucose was preferentially consumed over galactose during fed-batch cultivation. Preferential glucose uptake has been widely reported and is generally attributed to more efficient transport and catabolic integration of glucose into central metabolism compared to galactose, which requires additional enzymatic steps before entering glycolysis (Morlino et al. 2023). This consumption pattern confirms the effectiveness of the enzymatic strategy adopted and supports the feasibility of converting lactose-rich dairy by-products into fermentable substrates without relying on recombinant strains.

Supplementation of mRCEW with a coffee oil extract (EOC) markedly influenced cultivation performance. Data demonstrate that EOC supplementation predominantly stimulated biomass formation rather than increasing the intracellular PHB fraction. In shake-flask cultures, EOC addition resulted in a substantial increase in dry cell weight and absolute PHB concentration (g L⁻^1^), while the percentage of PHB relative to cell dry matter progressively decreased with increasing EOC concentrations. At 0.5% (v/v) EOC, the highest PHB titer was obtained, whereas higher supplementation levels led to further biomass increases accompanied by lower intracellular PHB contents.

This behavior is consistent with literature reports describing lipid-based substrates as effective growth-promoting carbon sources for *C. necator*, particularly when supplied in excess (Morlino et al. 2023; Weldon and Euler 2025). Under such conditions, carbon flux is preferentially directed toward biomass formation and maintenance metabolism rather than storage polymer synthesis, especially when nitrogen availability is not sufficiently limiting. This effect may be related to the typical composition of coffee oil extracted from spent coffee grounds, which is reported to contain mainly long-chain fatty acids such as linoleic, palmitic, oleic, and stearic acids, together with minor unsaponifiable compounds. Such lipid fractions can be efficiently converted into acetyl-CoA and reducing equivalents, thus supporting microbial growth and PHA biosynthesis (Jiang et al. 2011; Bhatia et al. 2018). However, the chemical composition of the coffee oil extract used in the present study was not experimentally determined and therefore the contribution of specific fatty acids or minor bioactive compounds to the observed physiological responses cannot be unequivocally established. The decline in intracellular PHB fraction became particularly evident at the highest EOC concentration tested (1.5% v/v, corresponding to approximately 15 g L⁻^1^), suggesting that excessive lipid availability may alter carbon partitioning between biomass formation and storage metabolism, thereby limiting intracellular PHB accumulation. Similar shifts in metabolic flux allocation have been reported for *Cupriavidus necator* cultivated on carbon-rich substrates, where conditions favoring rapid growth can reduce the proportion of carbon directed toward PHA storage (Morlino et al. 2023). Likewise, decreases in intracellular PHA accumulation at excessive lipid concentrations have been reported for other waste-derived oil supplements, indicating the existence of an optimal concentration window for maximizing polymer production (Bellini et al. 2022; Scott et al. 2021). Although absolute PHB concentrations were lower than those reported in some oil-based fed-batch systems using defined media (Nygaard et al. 2021; Bellini et al. 2022; Wang et al. 2012), the intracellular polymer fraction achieved here is remarkably high, particularly considering the use of a complex waste-derived substrate and a non-recombinant strain.

In addition, coffee-derived oils are reported to contain bioactive compounds. Although their contribution was not evaluated in the present study, such compounds may potentially influence microbial physiology (Hectors et al. 2025) and deserve further investigation. Importantly, the decrease in PHB content observed at the highest EOC concentration tested (1.5% v/v) suggests that excessive lipid loading may introduce metabolic imbalance or inhibitory effects, a phenomenon frequently observed when waste-derived supplements exceed an optimal concentration window (Morlino et al. 2023).

A central and distinctive aspect of this work is the evaluation of controlled oxidative stress as a process lever to enhance PHB production. The use of exogenous stressors to modulate PHA biosynthesis has been previously explored, most notably by Obruca et al. (2010b), who demonstrated that mild oxidative stress can increase PHB accumulation in *C. necator* when carefully timed and dosed. Importantly, their work showed that both ethanol and hydrogen peroxide affect the PHB biosynthetic pathway primarily by increasing the activity of upstream enzymes (namely β-ketothiolase and acetoacetyl-CoA reductase) while leaving PHB synthase activity largely unchanged. This suggests that oxidative stress does not directly stimulate polymerization but rather enhances precursor supply and redox balance, thereby favoring carbon flux toward PHB synthesis (Obruca et al. 2010a, b).

Ethanol and hydrogen peroxide, while both acting as oxidative stressors, exert their effects through partially distinct cellular mechanisms. Ethanol can perturb membrane integrity and alter intracellular redox balance, indirectly stimulating adaptive responses that increase flux through PHB precursor-generating reactions. Hydrogen peroxide, on the other hand, directly generates reactive oxygen species and activates oxidative stress response systems, leading to a reprogramming of cellular metabolism toward protective and storage functions. In both cases, when applied at sub-lethal concentrations, these stressors can promote PHB accumulation as part of a broader cellular strategy to cope with redox imbalance and environmental perturbation (Obruca et al. 2010b, 2021).

In the present study, oxidative stress induction at 24 h consistently resulted in better outcomes than supplementation at 60 h, both in terms of PHB accumulation and, in some cases, biomass formation. This timing-dependent effect can be interpreted in light of cellular physiological state. At 24 h, cells are still metabolically active and capable of mounting adaptive stress responses without severe growth impairment. Under these conditions, mild oxidative stress may induce a form of physiological “priming,” improving metabolic efficiency and robustness and, in turn, supporting increased biomass formation despite the presence of stress. In contrast, stress applied at 60 h likely coincides with nutrient limitation and a more fragile physiological state, where additional oxidative pressure exceeds adaptive capacity and results in growth inhibition. The observation that biomass can increase despite oxidative stress is consistent with the concept of hormesis, whereby low-level stress elicits beneficial adaptive responses rather than damage, as previously discussed for PHB production in *C. necator* (Obruca et al. 2010b). In contrast, the moderate decrease in biomass observed between 72 and 120 h is consistent with stationary-phase metabolic rearrangements typically reported under nutrient-limited and stress-modulated conditions, where intracellular PHB accumulation may continue despite partial biomass reduction (Obruca et al. 2010b; Morlino et al. 2023).

The fed-batch experiments further highlight the effectiveness of combining substrate management with stress modulation under controlled bioreactor conditions. Compared to batch operation, fed-batch cultivation resulted in higher final biomass and PHB concentrations, reflecting prolonged carbon availability and sustained metabolic activity. Repeated lactose hydrolysis following RCEW feeding ensured continuous supply of fermentable sugars, while preferential glucose consumption maintained efficient carbon flux toward central metabolism and PHB synthesis (Obruca et al. 2021). Other microbial systems evaluated on RCEW, such as *Haloferax mediterranei*, achieved approximately 1.18 g L⁻^1^ PHBV, including extensive membrane fractionation and enzymatic treatment (Raho et al. 2020). Our approach offers competitive PHB titers with substantially simpler upstream processing (Pagliano et al. 2020).

Finally, the comparison between conventional chloroform-based extraction and a chlorine-free PHB recovery method represents an important step toward improving the overall sustainability of the process. Based on the protocol described by Salvatori et al. (2023), which consists of alkaline hydrolysis followed by hydrogen peroxide oxidation, it is possible to effectively recover the polymer with high purity without the use of chlorinated solvents. In particular, PHB recovery obtained via the chlorine-free protocol was comparable to, or slightly higher than, that achieved with conventional extraction, confirming the effectiveness of this solvent-free strategy for polymer isolation from PHB-rich biomass produced from RCEW. Given that Salvatori et al. (2023) demonstrated improved recovery efficiency and polymer quality at increasing intracellular PHA contents, the high PHB fractions achieved here further support the suitability of chlorine-free extraction as a sustainable downstream option. Taken together, these results indicate that the integration of waste-derived substrates, mild stress modulation, and solvent-free downstream processing can significantly enhance the environmental and practical feasibility of PHB production using *C. necator*.

In addition to production performance, the physicochemical properties of the PHB obtained under fed-batch conditions provide important information on polymer quality and process robustness. The polymer recovered in this study consisted exclusively of 3-hydroxybutyrate units (100% 3HB), confirming the synthesis of a PHB homopolymer, as typically reported for *C. necator* cultivated on carbohydrate- or lipid-based substrates (Morlino et al. 2023; Weldon and Euler 2025).

The weight-average molecular weight (Mw) of approximately 876 kDa indicates the formation of a high–molecular weight PHB comparable to values reported for *Cupriavidus necator* cultivated under optimized nutrient-limited or fed-batch conditions. The polydispersity index (PDI = 1.90) reflects a moderately broad molecular weight distribution, which is commonly observed in PHB produced from complex waste-derived substrates and under stress-modulated cultivation regimes (Morlino et al. 2023; Hectors et al. 2025). The observed molecular weight distribution is consistent with PHB biosynthesis under dynamic cultivation conditions involving complex substrates and controlled oxidative stress, which can influence chain elongation kinetics and intracellular polymer turnover. High–molecular weight PHB is generally associated with improved mechanical performance and processability, further supporting the suitability of the proposed process for industrial-scale biopolymer production.

The industrial relevance of the proposed process is supported by the large availability of both feedstocks in Italy. Recent estimates indicate that RCEW, generated during ricotta manufacture, accounts for almost one million tons annually in Italy, representing a largely underutilized dairy side stream with significant environmental impact if improperly disposed of (Zilio and Vasmara 2025). In addition, coffee-processing residues are continuously generated by the Italian coffee roasting and consumption sectors and have been identified as promising substrates for biopolymer production and other biorefinery applications (Bhatia et al. 2018). The simultaneous valorization of these abundant agro-industrial residues supports the potential industrial applicability of the proposed PHB production process.

Overall, these results demonstrate that the proposed process not only enables efficient PHB production but also yields a polymer with physicochemical characteristics consistent with industrially relevant PHB grades reported in the literature.

## Conclusions

This study demonstrated the feasibility of producing poly(3-hydroxybutyrate) (PHB) by *Cupriavidus necator* DSM 428 using ricotta cheese exhausted whey (RCEW) as the main carbon source within a circular bioeconomy framework. The use of a mixed thermally conditioned substrate (mRCEW) provided a suitable nutritional balance for microbial growth and polymer accumulation, while coffee oil extract acted primarily as a growth-promoting co-substrate, increasing biomass formation and overall PHB productivity. In contrast, oxidative stress induced by hydrogen peroxide proved more effective in enhancing intracellular PHB accumulation, resulting in PHB contents exceeding 80% of cell dry matter under optimized conditions.

Trials performed in a controlled bioreactor further confirmed the robustness of the process, with fed-batch cultivation improving both biomass formation and PHB production. The resulting polymer exhibited physicochemical properties comparable to those reported for high-quality PHB produced from conventional substrates.

Future research should focus on a detailed characterization of coffee oil extracts and the metabolic fate of their individual components, as well as on the integration of lipid supplementation and oxidative stress strategies within optimized fed-batch processes. The implementation of controlled nitrogen-pulse or carbon/nitrogen co-feeding strategies may also represent a promising approach to optimize the balance between biomass formation and intracellular PHB accumulation, thereby further enhancing overall PHB productivity. Additional studies should also evaluate process performance at larger scales and assess the techno-economic and environmental sustainability of the proposed approach.

Overall, the proposed strategy demonstrates how two abundant agro-industrial side-streams, ricotta cheese exhausted whey and coffee-derived residues, can be integrated into an efficient PHB production process within a circular bioeconomy framework.

## Supplementary Information

Below is the link to the electronic supplementary material.

Supplementary Material 1 (DOCX 18.0 KB)

## Data Availability

No datasets were generated or analysed during the current study.
