# Supplementary material for: Poly(3-hydroxybutyrate) production from ricotta whey by Cupriavidus necator: effects of coffee oil and oxidative stress
Source: Appl Microbiol Biotechnol. 2026 Jun 26;110:267. doi: 10.1007/s00253-026-13933-8 (PMC13554019; doi:10.1007/s00253-026-13933-8)
Supplement: Supplementary file 1 [file 253_2026_13933_MOESM1_ESM.docx]

**Supplementary material.**

**Table 1S.** Kinetic parameters of lactose hydrolysis, sugar utilization, biomass formation, and PHB accumulation during fed-batch cultivation of *Cupriavidus necator* DSM 428

| **T (h)** | **Lactose**  **g L^-1^** | **Glucose**  **g L^-1^** | **Galactose**  **g L^-1^** | **Dry Cell Weight**  **g L^-1^** | **PHB**  **g L^-1^** | **% PHB/DCW** |
| --- | --- | --- | --- | --- | --- | --- |
| **0** | 38.1 ± 0.6a | 0.1± 0.2e | 0.1± 0.1d | - | - | - |
| **6** | 0.5 ± 0.2d | 12.0 ± 0.8a | 18.0 ± 1.0a | 0.10 ± 0.05h | 0.00 ± 0.01i | - |
| **12** | 0.5 ± 0.2d | 5.2 ± 0.5b | 8.6 ± 0.5c | 0.7 ± 0.2g | 0.03 ± 0.10i | 5.0 ± 0.5i |
| **18** | 0.3 ± 0.1d | 1.2 ± 0.3d | 7.8 ± 0.6c | 2.0 ± 0.2f | 0.18 ± 0.05i | 9.0 ± 0.8h |
| **24 pre-feed** | 0.2 ± 0.1d | 0.1 ± 0.2e | 7.14 ± 0.8c | 3.1 ± 0.3d | 0.44 ± 0.08g | 14.2 ± 0.6g |
| **24 post-feed** | 12.5 ± 0.3b | 0.07 ± 0.15e | 4.76 ± 0.55 | 2.07 ± 0.20f | 0.29 ± 0.05h | 14.2 ± 0.6g |
| **30** | 1.0 ± 0.2c | 4.8 ± 0.4b | 12.0 ± 0.6b | 2.6 ± 0.2e | 0.45 ± 0.06g | 17.3 ± 0.8f |
| **36** | 0.4 ± 0.1d | 2.5 ± 0.3c | 14.5 ± 0.4b | 3.4 ± 0.2d | 0.95 ± 0.07f | 27.9 ± 0.9e |
| **42** | 0.3 ± 0.1d | 1.0 ± 0.2d | 14.0 ± 0.3b | 4.1 ± 0.2c | 1.60 ± 0.08e | 39.0 ± 1.0d |
| **48** | 0.2 ± 0.1d | 3.4 ± 0.3b | 13.4 ± 0.2b | 4.65 ± 0.10b | 2.25 ± 0.09d | 48.5 ± 1.1c |
| **54** | 0.15 ± 0.10d | 1.5 ± 0.2d | 12.0 ± 0.3b | 4.80 ± 0.15a | 2.90 ± 0.10c | 60.4 ± 1.0b |
| **60** | 0.12 ± 0.10d | 0.9 ± 0.2d | 10.5 ± 0.c | 4.90 ± 0.20a | 3.30 ± 0.11b | 67.3 ± 0.8a |
| **66** | 0.11 ± 0.10d | 0.6 ± 0.1d | 9.6 ± 0.2c | 4.95 ± 0.30a | 3.45 ± 0.12b | 69.7 ± 0.7a |
| **72** | 0.1 ± 0.1d | 0.4 ± 0.1e | 8.7 ± 0.2c | 4.94 ± 0.55a | 3.56 ± 0.12a | 72.2 ± 0.6a |

^a-i^Values in the same column with different superscript letters are significantly different at P<0.05.
